# Early Presentation of a Rare Complication of Sodium-Glucose Cotransporter-2 Inhibitors 10 Days After Initiation: Case Report and Literature Review

**DOI:** 10.7759/cureus.5173

**Published:** 2019-07-19

**Authors:** Ghada Elshimy, Ricardo Correa, Mahmoud Alsayed, Sathya Jyothinagaram

**Affiliations:** 1 Endocrinology, Diabetes and Metabolism, University of Arizona College of Medicine-Phoenix, Phoenix, USA

**Keywords:** sodium-glucose co transporter-2 (sglt2) inhibitors, fournier's gangrene

## Abstract

Fournier’s gangrene is an extremely rare infection that can occur in immunocompromised patients, especially those with diabetes. Given the severity of this infection and the new associated link to sodium-glucose cotransporter-2 inhibitors, the US FDA recently issued a warning in August 2018. Few cases of Fournier’s gangrene have been reported in the literature in diabetic patients taking these oral medications. We report a case of Fournier’s gangrene presenting 10 days after a patient with type 2 diabetes started empagliflozin therapy.

## Introduction

Fournier’s gangrene is an extremely rare infection that can occur in patients with diabetes. The US FDA recently issued a warning about patients taking sodium-glucose cotransporter-2 (SGLT2) inhibitors in that these patients are liable to develop Fournier’s gangrene. This complication usually occurs a few months after starting the medication [1-12]. A total of 12 cases have been reported in the literature from May 2013 to May 2018 (Abstract: Chi WC, Lim-Tio S. Fournier’s Syndrome: A Life-Threatening Complication of SGLT2 Inhibition in Poorly Controlled Diabetes Mellitus. 2016 Joint Annual Scientific Meeting of the Australian Diabetes Educators Association (ADEA) and the Australian Diabetes Society (ADS); August 25, 2016) [1-3]. This number increased to 55 cases by January 31, 2019 as per Bersoff-Matcha et al. [8]. We report another case of Fournier’s gangrene associated with the use of empagliflozin. Our case is unique, given this rare complication occurred only 10 days after the patient began the medication without a prior history of genital or urinary infection.

## Case presentation

**Figure 1:**
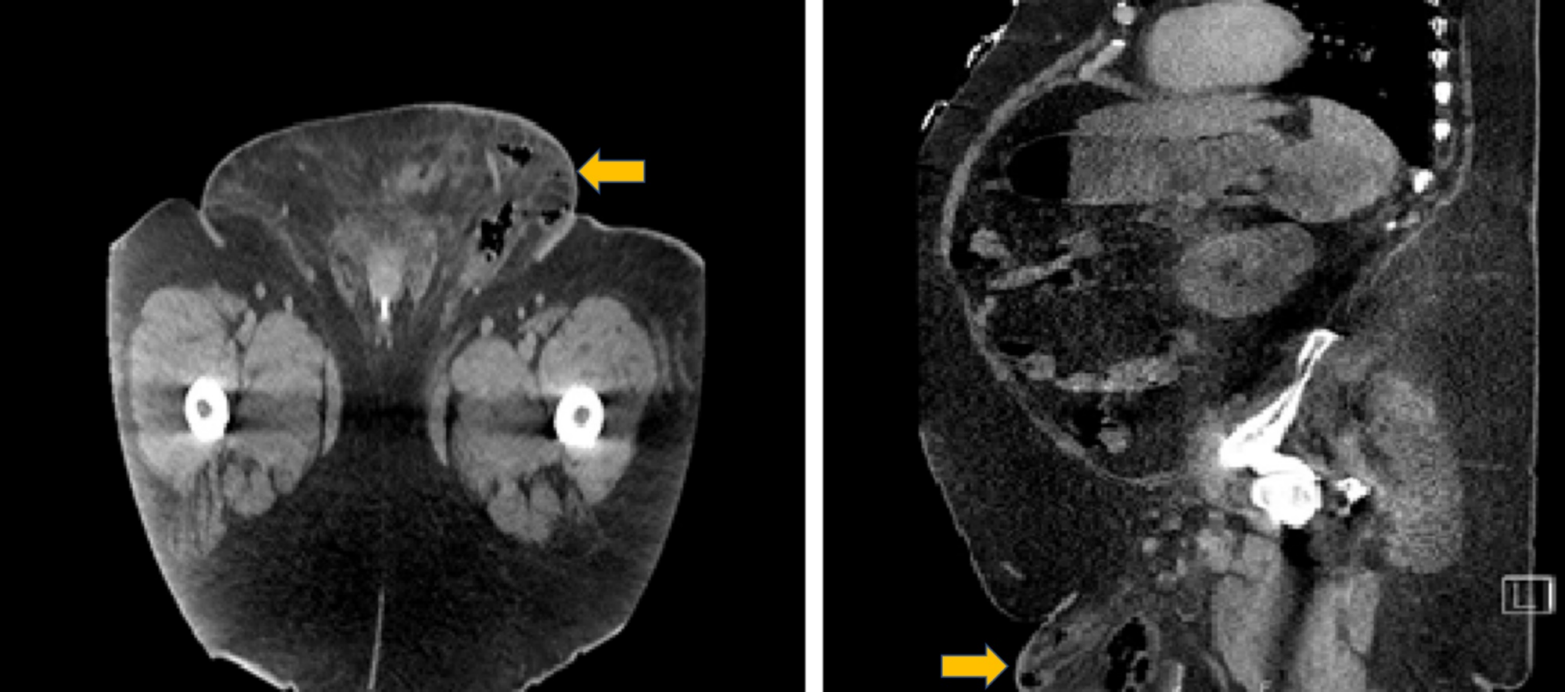
Abdominal CT showing gas in the subcutaneous tissue of the scrotum confirming the diagnosis of Fournier’s gangrene.

A 57-year-old white man, with a past medical history of uncontrolled type 2 diabetes diagnosed around 10 years ago and complicated by peripheral neuropathy, Hashimoto's hypothyroidism, and morbid obesity with no prior history of genital or urinary tract infection, was being treated as an outpatient by a private endocrinologist. His glycosylated hemoglobin levels were uncontrolled despite being on the following oral medications: glipizide 10 mg twice daily, metformin 1 gm twice daily, and linagliptin 5 mg daily. Therefore, his health care team decided to start him on additional oral medication for better control. Empagliflozin was added to his medication regimen. After 10 days, the patient reported concerns of severe left groin pain initially treated as cellulitis in an outpatient facility. Given no improvement in his condition on oral antibiotics, he decided to go to the emergency department. On physical examination, his weight was 192 kg with a BMI of 62.76 kg/m2. He was afebrile and vitally stable. Perineal examination revealed a grossly swollen and indurated right scrotum with tender spermatic cord, epididymis, and testicles. There was associated bilateral inguinal lymphadenopathy. Urine analysis showed +3 glucose confirming adherence to empagliflozin. A CT scan revealed Fournier’s gangrene (Figure 1).

The patient required two surgical interventions with hyperbaric oxygen therapy. An endocrinologist was consulted for inpatient diabetes management as our patient required a high dose of insulin after stopping all of his oral medications during the hospital course.

## Discussion

Several infections can occur in a patient with uncontrolled diabetes, including Fournier's gangrene and other life-threatening infections. SGLT2 inhibitors are known to increase the risk of genitourinary infections. Given the recent FDA warning regarding the new adverse side effect of SGLT2 inhibitors, physicians should be aware that Fournier's gangrene can occur in patients with diabetes, especially those with a potential risk of a genital infection (Abstract: Chi WC, August 25, 2016) [1-12]. Despite all the positive metabolic effects, reduction in cardiovascular events and delay in the progression of kidney disease of SGLT2 inhibitors, the risk of serious infections should be considered [13-14]. Some studies show that SGLT2 inhibitors raise the risk of developing genital infections and, to a relatively less extent, urinary tract infections due to the pharmacologically-induced glucosuria that promotes the growth of commensal genital microorganisms [15].

According to the FDA report, of the 12 cases of Fournier’s gangrene associated with SGLT2 inhibitor use, seven were men and five were women, and the gangrene developed several months after starting the medication. All 12 patients were hospitalized and required surgery, similar to our patient who had a complicated hospital course requiring two surgical interventions with hyperbaric oxygen and a high dose of insulin to control the blood glucose. This number increased to 55 cases by January 31, 2019 as per Bersoff-Matcha et al. [8]. It seems likely that additional cases exist but are as yet unreported in the literature. A recently published update on SGLT2 inhibitors by Scheen stated that these adverse events should not mask the overall cardiovascular and renal benefit of SGLT2, especially in patients at high cardiovascular risk [9].

Morbidly obese patients are more liable to get severe and recurrent genital infections. Studies showed that a high body mass index is associated with an increased risk for urinary tract infection and pyelonephritis [13]. Our patient was morbidly obese. However, he had no prior urinary tract infections or prior genital infections like the patient described by Onder et al. in 2019 [10]. However, the patient described by Kumar et al. had multiple genital thrush prior to starting empagliflozin [1]. Our patient is unique compared to other patients described in literature given the early presentation (only 10 days after starting empagliflozin). Time to onset after initiation of SGLT2-inhibitor therapy ranged from five days to 49 months as per Bersoff-Matcha et al., and the early onset cases were patients with prior urinary tract infections, unlike our patient [8]. Other patients described in the literature such as Chi’s patient presented three weeks after starting dapagliflozin (Abstract: Chi WC, August 25, 2016); Onder’s patient developed Fournier's gangrene six months after using dapagliflozin [10]. All these patients were morbidly obese, which is considered an additional predisposing factor.

## Conclusions

SGLT2 inhibitors have been commonly associated with urinary tract infections. However, diabetes and obesity may increase the risk of potentially more severe infections such as Fournier's gangrene. Despite all the positive metabolic effects, reduction in cardiovascular events and delay in the progression of kidney disease of SGLT2 inhibitors, the risk of serious infections should be considered. Physicians should be aware of this rare complication and assess each patient prior to prescribing SGLT2 inhibitors with specific caution in morbidly obese patients.
